# Temporomandibular joint morphology in Korean using cone-beam computed tomography: influence of age and gender

**DOI:** 10.1186/s40902-021-00307-5

**Published:** 2021-07-05

**Authors:** Ji-Min Yun, Young-Jun Choi, Su-Heon Woo, Ui-Lyong Lee

**Affiliations:** 1 Department of Orthodontics, Dental Center, Chung-Ang University Hospital, Seoul, Republic of Korea; 2 Department of Oral & Maxillofacial Surgery, Chung-Ang University College of Medicine, Seoul, Republic of Korea; 3 R&D Center, Medyssey Co, Ltd., Jecheon, Republic of Korea; 4 Department of Oral & Maxillofacial Surgery, Dental Center, Chung-Ang University Hospital, 224-1 Heukseok-dong, Dongjak-gu, Seoul, Republic of Korea

**Keywords:** Condyle morphology, Temporomandibular joint, Cone-beam computed tomography, TMJ prosthesis

## Abstract

**Objectives:**

The purpose of this study was to investigate the condylar morphology and position of Koreans using cone-beam computed tomography (CBCT) images. Analyzing the mean values of this study with reference to left and right sides, gender, and age will help to understand the size of the condyle and glenoid fossa, condylar morphology, and temporomandibular joint (TMJ) symmetry for establishing the standard temporomandibular joint structures of Koreans and then design the standard temporomandibular joint prosthesis for Koreans.

**Results:**

There was no significant result in the condyle size, condyle axis angle, joint space, fossa depth, and mandibular body size between the left and right sides (*p* > 0.05). On the other hand, the mediolateral width of the condyle and mandibular body size show significantly different with the gender (*P* < 0.05). Also, significant differences were found in condyle size, joint space, fossa depth, and mandibular body size according to age groups (*p* < 0.05).

**Conclusions:**

Condylar position and morphology vary according to side, age, and gender. The results of this study are expected to help in customizing a treatment for the patients who need TMJ reconstruction by predicting the TMJ morphology according to age and gender and design the standard temporomandibular joint prosthesis for the Koreans.

## Background

The temporomandibular joint (TMJ) is a joint formed between the mandibular condyle and the base of the cranial bone. This joint is essential for stable occlusion and mastication [1]. The TMJ component between the mandible and the cranial bone is thought to maintain remodeling capability even after growth completion [2]. In adulthood, the condyle undergoes a remodeling process that can affect the quantity and morphology due to adaptability, i.e., flatness, hardening, erosion, presence of bony spurs, and absorptions [3]. The continuously changing morphology is the process of adapting to functional and mechanical demands [4, 5]. In addition, several factors, including age, gender, pathologic process, functional changes, and bite forces, were found to affect TMJ morphology and position [6–8].

A radiologic examination may be helpful in the diagnosis of temporomandibular disorder (TMD) [1]. Diagnosis of TMD is complex and requires comprehensive clinical and radiographic analysis [9]. The complex structure of the TMJ makes it difficult to perform radiographic examinations, and an accurate diagnosis requires several types of radiographic images [10]. Conventional two-dimensional (2D) radiography had been the main tool to observe the TMJ. However, this 2D technique is not accurate owing to the overlap of neighboring structures, as well as the low sensitivity to changes in both condylar and temporal bone components [11, 12]. The advancement of three-dimensional (3D) imaging has enabled a much more accurate analysis of TMJ than ever before [13]. Cone-beam computed tomography (CBCT) has less radiation exposure compared to conventional computed tomography (CT), and its high-resolution imaging can achieve high levels of accuracy when evaluating the TMJ [14–17].

Although many patients with TMJ disorder are initially managed with non-surgical and conservative treatments, some patients who have disorders with pathological and physiological function at the end stage may need to undergo TMJ reconstruction [18]. Even though TMJ reconstruction using alloplastic prosthesis is a procedure that provides biomechanics, not a biological solution, for the treatment of severe joint diseases [19], alloplastic TMJ prosthesis can be applied to many indications, such as bony ankyloses, failure in the previous allograft and autogenous joint replacement, post-traumatic condylar injury, avascular necrosis, reconstruction after tumor resection, developmental abnormalities, functional abnormalities, and severe inflammation that does not respond to conservative treatment [18]. Successful TMJ prosthesis must meet several broad biological and mechanical properties. One of them is a simulation of functional TMJ motion. To mimic the motion of the joints, the patient-customized joint reproduction is important. The TMJ prosthesis currently used in Korea is made in the USA. Therefore, it is necessary to develop the Korean-type TMJ prosthesis.

Many efforts have been made to investigate the TMJ anatomy using multiplanar CT examination of the condyle. The position and size of the TMJ and clinical significance have always been controversial [20]. It is not easy to standardize the morphology, size, and relationship of the TMJ due to inherent diversity and TMJ’s continuous adaptation process according to time and pathological deformation. Although the size and position of the condyle were previously examined, there is still a lack of information regarding these, especially age factors, in the Korean population.

Therefore, the purpose of this study was to investigate the effect of left and right sides, gender, and age on the condylar morphology and position through CBCT images. Subsequently, this study analyzed the size of the condyle and glenoid fossa, condylar morphology, and TMJ symmetry relationship to study the standard temporomandibular joint structures of Koreans. And then based on the conclusions drawn in this study, it will be used as basic data to develop a TMJ prosthesis specialized for Koreans.

## Methods

### Participants

CBCT images of 240 adult patients (480 TMJs) who visited the Department of Oral and Maxillofacial Surgery of University Hospital from 2014 to 2016 were reviewed. CBCTs were taken for reasons such as diagnosis of lesions in the jaw, preparation for extraction of the third molar, diagnosis of available bone for implant placement, and preparation for orthognathic surgery. This study protocol was approved by the university hospital ethics review committee (IRB No. 1811-024-16230). The CBCT images were divided into three groups according to the age: group 1 (20–39 years old), group 2 (40–59 years old), and group 3 (above 60 years old). Each group included 80 persons (40 males and 40 females).

Patients who underwent orthognathic surgery, patients with skeletal anomalies such as craniofacial synostosis and facial cleft, and patients with more than 4-mm menton deviation were excluded. Before conducting the measurement, we checked the morphology of the mandibular condyle. Subjects with pathologic radiographic signs of condyle such as flattened surface, erosion, irregularities, subcondral cysts, and osteophytes were excluded. Tooth loss was not considered. We also did not consider the presence of systemic diseases such as hypertension, diabetes, infectious disease, and digestive tract diseases.

### CBCT analysis

**Fig. 1:**
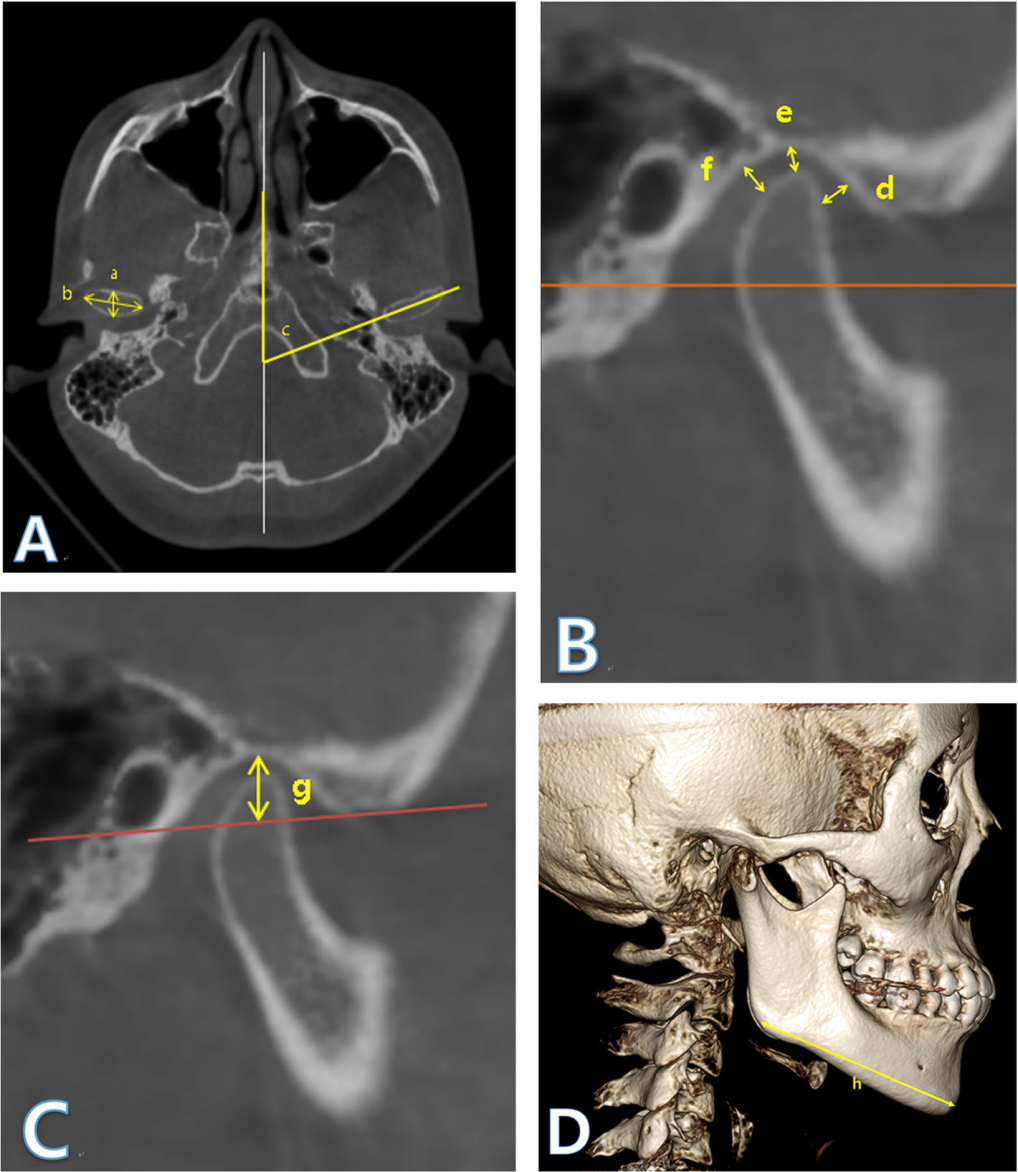
Measuring method on the In Vivo 5 dental software. (a) Anteroposterior width, (b) mediolateral width, and (c) condyle axis angle (**A**). (d) Anterior joint space, (e):superior joint space, and (f) posterior joint space (**B**). (g) Fossa depth (**C**). (h) Mandibular body size (**D**)

All CBCT images were obtained with the same CBCT instrument (3D eXam, Kavo Dental GmbH, Biberach, Germany). CT images included the entire maxilla and mandible, and images of all subjects were analyzed by the same dentist using the same machine. After converting the images to the Digital Imaging and Communication in Medicine (DICOM) format using the In Vivo 5 Dental software (Anatomage, San Jose, CA, USA), the joints were reconstructed in three dimensions and measured in multi-planar reconstruction. The left and right TMJs were independently assessed for all subjects. To equalize the reference plane for the evaluation, both eyes and zygoma were placed on a straight line in the axial plane. A difference of 6° from the SN plane (Sella–Nasion plane) was applied to the sagittal plane to adjust it to the Frankfort horizontal (FH) plane. On the coronal plane, the crista gali and anterior nasal spine points were placed on the vertical line to adjust the left and right sides. The size of the condyle and the angle of the condyle axis were measured at the point where the width of the condyle was widest on the axial plane (Fig. 1A (a–c)). The position of each condyle was determined by measuring the space between the anterior, upper, and posterior joints (Fig. 1B (d–f)), and the depth of the articular tubercle was measured to determine the fossa morphology (Fig. 1C (g)). The intraarticular space and the depth of the articular tubercle were measured on the sagittal plane corresponding to the midpoint of the selected axial view of the condyle. The length of the mandibular body was measured as the distance between the gonion and the menton in the 3D reconstructed image (Fig. 1D (h)). The markers used in the analysis are shown in Table 1 and set in the following manner.

**Table 1:** The markers used in the analysis

Measurement	Definition
Antero-posterior width (mm)	The anteroposterior diameter of the condylar process
Medio-lateral width (mm)	The mediolateral diameter of the condylar process
Condyle axis angle (°)	Angle between the medio-lateral plane of the condylar process and the midsagittal plane
Anterior joint space (mm)	The shortest distance between the posterior wall of the articular tubercle and the most anterior point of the condylar head
Superior joint space (mm)	The distance between the most superior point of the mandibular fossa and the most superior point of the condylar head
Posterior joint space (mm)	The shortest distance between the posterior wall of the mandibular fossa and the most posterior point of the condylar head
Depth of mandibular fossa (mm)	The distance between the most superior point of the mandibular fossa and the plane formed by the most inferior points of the articular tubercle and the external meatus
Size of mandibular body (mm)	The distance between the menton and gonion

### Statistical analysis

The measurements were processed and analyzed using SPSS 21.0. The paired-sample t test was used to calculate the statistically significant differences of condyle size, degree, joint space, fossa depth, and mandibular body size between left and right sides, and the t test for independent samples was employed to calculate the statistically significant differences between the males and the females. An analysis of variance (ANOVA) was performed to do the analyses based on ages. The Bonferroni method was used for comparison of two of the three age groups. The level for significance was set at *P* < 0.05.

## Results

Each mean value divided into groups and gender is shown in Tables 2, 3, and 4, and it was rounded up in the second decimal place. The parenthesized values are the standard deviation values.

**Table 2:** Table comparing left and right based on gender

	Sex	Mean		*p*-value	Mean difference
		Right (*n* = 240)	Left (*n* = 240)		
Anteroposterior width	Male (*n* = 120)	8.18 (1.5)	8.15 (1.8)	.895	.02833
	Female (*n* = 120)	8.00 (1.5)	7.85 (1.4)	.383	.15250
Mediolateral width	Male	20.97 (3.0)	20.52 (2.7)	.181	.44417
	Female	18.52 (2.3)	17.99 (2.9)	.117	.53167
Condyle axis angle	Male	70.53 (8.2)	71.69 (9.0)	.284	− 1.15500
	Female	70.95 (9.1)	72.23 (7.9)	.245	− 1.28500
Anterior joint space	Male	2.10 (1.0)	2.07 (0.8)	.771	.03233
	Female	1.91 (0.8)	2.00 (0.7)	.366	− .09017
Superior joint space	Male	3.75 (1.2)	3.84 (1.4)	.56	− .09442
	Female	3.58 (1.5)	3.50 (1.4)	.671	.07917
Posterior joint space	Male	2.41 (1.2)	2.53 (1.3)	.454	− .12417
	Female	2.31 (1.1)	2.26 (1.2)	.753	.04717
Fossa depth	Male	9.09 (2.3)	9.33 (2.6)	.449	− .23967
	Female	9.35 (3.1)	9.33 (3.3)	.956	.02242
Mandibular body	Male	89.51 (6.3)	89.07 (5.8)	.579	.43392
	Female	83.92 (7.0)	83.93 (7.1)	.996	− .00483

**Table 3:** Table comparing male and female based on left and right side

	Side	Mean		*p*-value	Mean difference
		Male (*n* = 120)	Female (*n* = 120)		
Anteroposterior width	Right (*n* = 240)	8.18 (1.5)	8.00 (1.5)	.319	.18167
	Left (*n* = 240)	8.15 (1.8)	7.85 (1.4)	.143	.30583
Mediolateral width	Right	20.97 (3.0)	18.52 (2.3)	.000	2.44417
	Left	20.52 (2.7)	18.00 (2.9)	.000	2.53167
Condyle axis angle	Right	70.53 (8.2)	70.95 (9.1)	.708	− .41667
	Left	71.69 (9.0)	72.23 (7.9)	.609	− .54667
Anterior joint space	Right	2.10 (1.0)	1.91 (0.8)	.092	.19058
	Left	2.07 (0.8)	2.00 (0.7)	.487	.06808
Superior joint space	Right	3.75 (1.2)	3.58 (1.5)	.328	.16992
	Left	3.84 (1.4)	3.50 (1.4)	.055	.34350
Posterior joint space	Right	2.41 (1.2)	2.31 (1.1)	.526	.09567
	Left	2.53 (1.3)	2.26 (1.2)	.106	.26700
Fossa depth	Right	9.09 (2.3)	9.35 (3.1)	.453	− .26308
	Left	9.33 (2.6)	9.33 (3.3)	.998	− .00100
Mandibular body	Right	89.51 (6.3)	83.92 (7.0)	.000	5.58308
	Left	89.07 (5.8)	83.93 (7.1)	.000	5.14433

**Table 4:** Table comparing gender and right/left side based on ages and Bonferroni test of dependent variable among different age groups

	Side	Mean			*p*-value	Mean			*p-*value
		Male group (*n* = 120)				Female group (*N* = 120)			
		1 (*N* = 40)	2 (*N* = 40)	3 (*N* = 40)	Male	1 (*N* = 40)	2 (*N* = 40)	3 (*N* = 40)	Female
Anteroposterior width	Right	8.0 (1.3)	8.0 (1.7)	8.5 (1.4)	.317	7.3 (1.5)	7.9 (1.1)	8.7 (1.5)^a,b^	.000
	Left	7.6 (1.8)	8.1 (1.7)	8.2 (1.9)^a^	.027	7.3 (1.2)	7.8 (1.5)	8.4 (1.2)^a^	.002
Mediolateral width	Right	19.4 (3.7)	21.3 (2.7)^a^	21.7 (1.8)^a^	.002	18.0 (2.1)	19.0 (2.8)	18.6 (2.0)	.216
	Left	19.6 (2.7)	21.1 (2.8)^a^	20.9 (2.4)	.030	17.6 (2.5)	17.9 (3.4)	18.5 (2.7)	.342
Condyle axis angle	Right	70.1 (7.8)	69.2 (6.9)	72.5 (9.6)	.212	68.1 (9.9)	71.7 (8.0)	73.1 (8.9)^a^	.039
	Left	70.5 (10.4)	71.3 (7.1)	73.8 (9.0)	.207	69.7 (8.4)	71.5 (6.5)	75.5 (7.6)^a^	.003
Anterior joint space	Right	2.0 (1.1)	2.2 (0.7)	2.0 (1.1)	.840	2.0 (0.9)	1.7 (0.6)	2.0 (0.9)	.193
	Left	1.9 (0.7)	2.4 (0.9)^a^	1.9 (0.7)	.020	2.1 (0.9)	1.9 (0.6)	2.0 (0.7)	.670
Superior joint space	Right	3.1 (0.8)	4.0 (1.1)^a^	4.2 (1.2)^a^	.000	3.6 (1.7)	3.5 (1.4)	3.6 (1.4)	.931
	Left	3.2 (1.3)	4.0 (1.2)^a^	4.3 (1.4)^a^	.001	3.4 (1.6)	3.4 (1.3)	3.7 (1.2)	.690
Posterior joint space	Right	2.1 (0.6)	2.5 (1.1)	2.7 (1.7)	.111	2.1 (0.9)	2.2 (1.0)	2.6 (1.3)	.078
	Left	2.0 (0.7)	2.7 (1.2)	2.9 (1.7)^a^	.005	1.9 (0.8)	2.2 (0.9)	2.6 (1.7)^a^	.042
Fossa depth	Right	9.7 (3.2)	9.1 (1.4)	8.4 (1.7)^a^	.043	10.5 (4.3)	9.3 (2.2)	8.2 (1.8)^a^	.005
	Left	10.1 (3.8)	9.1 (1.7)	8.8 (1.5)	.078	10.8 (4.3)	9.1 (2.6)	8.1 (1.8)^a^	.001
Mandibular body	Right	91.4 (7.6)	88.2 (6.0)	88.9 (4.5)	.055	86.7 (8.4)	83.4 (6.0)	81.6 (5.3)^a^	.004
	Left	90.8 (7.0)	87.3 (5.5)^a^	89.1 (4.1)	.028	87.0 (8.4)	83.0 (6.0)^a^	81.8 (5.6)	.002

^a^*P* < 0.05 (compare with group 1)

^b^*P* < 0.05 (compare with group 2)

### Comparisons between left and right sides

There was no significant difference in condylar size, angle, joint space, fossa depth, and mandibular body in the left and right (*P* > 0.05). It means that left and right TMJs are symmetric (Table 2).

### Comparisons between males and females

The mediolateral width of the condyle and the size of the mandibular body showed a significant difference according to gender (*P* < 0.05). Men had larger mediolateral width and mandibular body size compared to women (Table 3).

### Comparisons among different age groups

#### Differences in condyle morphology

There was a significant difference in condyle size according to age (*P* < 0.05). When three age groups were compared, mediolateral width for males and anteroposterior width and condyle axis angle for females were significant in both left and right sides (Table 4).

The Bonferroni test was performed to evaluate the significance between the groups. In males, there was a statistical significance between groups 1 and 2 and between groups 1 and 3 in the right mediolateral width and between groups 1 and 2 in the left mediolateral width. In females, there was a significant difference between groups 1 and 3 and between groups 2 and 3 in the right anteroposterior width, between groups 1 and 3 in left anteroposterior width, and between groups 1 and 3 in condyle axis angle in left and right (*P* < 0.05) (Table 4).

#### Differences of the condylar position among different age

There was a significant difference in joint space and fossa depth according to age (*p* < 0.05). Males were statistically significant in the left and right superior joint spaces, the left anterior joint space, and the left posterior joint space, and females showed significant values at the left and right fossa depths (Table 4).

In the Bonferroni test, the superior joint space of the male group was significantly different between groups 1 and 2 and between groups 1 and 3. Also, in males, there were significant differences in the left anterior joint space between groups 1 and 2, left posterior joint space between groups 1 and 3, and right fossa depths between groups 1 and 3. In females, there were statistically significant differences in the left and right fossa depths between groups 1 and 3 (Table 4).

Thus, the location of the condyle can be influenced by the factors of age.

#### The change of mandibular body

There was a significant difference in mandibular body size according to age (*p* < 0.05) (Table 4).

The Bonferroni test showed a significant difference between groups 1 and 3 in the right side and between groups 1 and 2 and groups 1 and 3 in the left side (Table 4).

## Discussion

The TMJ is a part of the configuration of the maxillofacial function system along with the teeth, maxillofacial bone, masticatory muscle, nerves, and blood vessels. Assessing the anatomical structure of the TMJ is an important way to study its morphology and function and will help in the diagnosis, treatment, and evaluation of TMJ disease. This study randomly selected patients who visited our hospital and underwent CBCT from 2014 to 2016.

In general, the mandibular condyle is observed as having a crescent-shaped elliptical articular surface, and the mediolateral width is twice as long as the anteroposterior width. The angle of the condyle is rotated about 20° inward on average. The morphology of the condyle is diverse, and vertical and anterior osteophyma is often observed. The beak-shaped anterior osteophyte appears to coincide with the area where excessive extension or load is applied. Flattening and erosion of the condyle articular surface seem to be evidence of an ischemic response similar to avascular necrosis and are caused by a mechanical overload on the joints. In fact, some patients clearly showing arthritis and osteophyma were included in the CBCT image.

CBCT is a powerful tool for diagnosing TMD [9, 17, 21]. The CBCT scan provides multiplanar images of the condyle and surrounding structures, which are reconstructed in three dimensions to analyze the TMJ morphology, position, and dynamics [14, 22–24]. This enables easier and more accurate visualization of complex anatomical structures with less radiation exposure, lower operating costs, and reduced scan times compared to the traditional helical CT [14, 23, 25–27]. Thus, when used properly, CBCT imaging can present more accurate and valuable diagnostic information compared to conventional radiography techniques [28, 29].

In this study, all variables were measured using CBCT software. The software simultaneously provides sagittal, axial, coronal, and 3D reconstruction views of all landmarks. Thus, the measurement errors due to incorrect determination of critical anatomical points were minimized [21].

### Difference in condylar morphology

The subjects enrolled in this study did not show any discomfort of TMD, so the measured variables also did not show any significant results. This shows that the sizes and angles of the condyles are basically symmetric between the left and right sides. There are various factors that determine the asymmetry of the TMJ structure, such as the absence of teeth, tooth wear, premature occlusal contact point, and functional deviation of the mandible. Articular cartilage is known to respond to degenerative changes and non-physiological deformities of the joints by changing the single cartilage layer and total layer thickness, which will lead to a change in vertical length and width [30].

In our study, the mediolateral width of a man’s left and right condyle was significantly higher than that of a woman’s left and right condyle. The difference in condylar morphology according to gender should be consistent with the difference in skeletal characteristics, which is similar to Song et al.’s result stating that the frontal and lateral facial measurements in men were greater than those in women [31].

Significant differences were found in condylar morphology according to age groups. In men, the mediolateral width of the left and right condyles increased with age, and this trend was evident when comparing groups 2 and 3 with group 1. Condyles were more likely to be reconstructed at the age of 40 years or older than at the age of 20–30 years, and it was also possible to infer the morphology change pattern. Unlike in men, the anteroposterior width and condyle axis angle in females significantly increased with age. In terms of condylar morphology, older individuals were more likely to exhibit degenerative changes than young individuals [20]. Therefore, the change in condyle size was more apparent when compared with group 1. There is a significant relation between disk displacement without reduction and degenerative bone changes in the temporomandibular joint [32]. So, CT images taken for the diagnosis of TMD were not included in this study. And we excluded the patients who have TMD symptoms.

### Differences in condylar position

As regards the condylar morphology, there was no significant result in the joint space between the left and right sides and the condylar position of the fossa depth. Therefore, this result suggests that the condylar position is also symmetric. There was no difference in condyle position according to gender. Some variables showed significant differences in the comparison of the condylar position according to age. The length of the superior joint space of the left and right sides in men tended to increase gradually with increasing age, and this trend was also evident when comparing groups 2 and 3 with group 1. The fossa depth of the left and right sides in women also showed significant results, and the depth decreased with age. The difference was obvious when comparing group 1 with group 3. Aging may have caused the flattening of the superior part of the condyle and erosion of the articular tubercle. Therefore, the position of the condyle may change with age.

### Differences in the mandibular body

The size of the mandibular body did not show significant results when comparing the right and left sides, which suggests that the mandibular body is generally symmetric. The mandibular body size was significantly higher in men than in women (*p* < 0.05). In women, the distance from the menton to the gonion tended to decrease with age. In both the left and right sides, there was a clear difference between group 1 (20–30 years old) and group 3 (60 years old or older), which may have resulted from the effect of bone remodeling due to tooth loss.

### Application to allosteric TMJ

In this study, the morphology, position, and size of the temporomandibular joints of Koreans, according to the left and right sides, gender, and age, were inferred using subjects aged 20 to 88 years. Alloplastic TMJ is a procedure that provides biomechanics, not a biological, solution for the treatment of severe joint diseases [19]. To mimic the motion of the joints, the patient-customized joint reproduction is of utmost importance. Indications for alloplastic TMJ include bony ankyloses, failure in previous allograft and autogenous joint replacements, post-traumatic condylar injury, avascular necrosis, reconstruction after tumor resection, developmental abnormalities, functional abnormalities, and severe inflammation that does not respond to conservative treatment [18]. The older the patient, the more likely alloplastic TMJ is to be applied, but young patients may also undergo alloplastic TMJ for treatment of trauma and dysfunction. Analyzing the mean values of this study with reference to gender and age will help to reproduce the appropriate TMJ structure.

## Conclusion

The present study showed symmetry in condyle morphology, condyle position, and mandibular body size between the left and right sides, and no specific directionality was observed. In terms of gender, the mediolateral width and mandibular body size of the left and right condyle were greater in men than in women. In terms of age, the mediolateral width and superior joint space of the left and right condyle in men and the anteroposterior width, condyle axis angle, fossa depth of the left and right condyle, and mandibular body size of the left and right side in women showed significantly different results.

In conclusion, there was no significant difference, as condylar morphology, position, and mandibular body size were symmetric between the left and right sides. Gender and age factors seemed to have a certain influence on the morphology and position of the condyle. This information may be clinically useful to establish diagnostic criteria for the condylar morphology and position of Koreans. This study analyzed the changing pattern of the TMJ according to age and gender and measured the mean size and position. The results of this study are expected to help in customizing a treatment for each patient by predicting the TMJ morphology according to the patient during the allosteric TMJ surgery in Korea.

## Data Availability

All data and materials are available.

## Abbreviations

CBCT: Cone-beam computed tomography
TMJ: Temporomandibular joint
DICOM: Digital Imaging and Communication in Medicine
TMD: Temporomandibular disorder
FH plane: Frankfort horizontal plane
